# The nuclear sirtuin SIRT6 protects the heart from developing aging-associated myocyte senescence and cardiac hypertrophy

**DOI:** 10.18632/aging.203027

**Published:** 2021-05-02

**Authors:** Vinodkumar B. Pillai, Sadhana Samant, Samantha Hund, Madhu Gupta, Mahesh P. Gupta

**Affiliations:** 1 Department of Surgery, Basic Science Division, The Pritzker School of Medicine, University of Chicago, Chicago, IL 60637, USA

**Keywords:** cardiac aging, Sirt6

## Abstract

Sirtuins have been shown to regulate the aging process. We have previously demonstrated that Sirt6 blocks the pressure overload-induced cardiac hypertrophy in mice. Here, we show that Sirt6 can also mitigate aging-induced cardiomyocyte senescence and cardiac hypertrophy. We found that aging is associated with altered Sirt6 activity along with development of cardiac hypertrophy and fibrosis. Compared to young mice (4-months), the hearts of aged mice (24-months) showed increased levels of mitochondrial DNA damage, shortened telomere length, and increased accumulation of 8-oxo-dG adducts, which are hallmarks of aging. The aged hearts also showed reduced levels of NAD^+^ and altered levels of mitochondrial fusion-fission proteins. Similar characteristics were observed in the hearts of Sirt6 deficient mice. Additionally, we found that doxorubicin (Dox) induced cardiomyocyte senescence, as measured by expression of p16^INK4a^, p53, and β-galactosidase, was associated with loss of Sirt6. However, Sirt6 overexpression protected cardiomyocytes from developing Dox-induced senescence. Further, compared to wild-type mice, the hearts of Sirt6.Tg mice showed reduced expression of aging markers, and the development of aging-associated cardiac hypertrophy and fibrosis. Our data suggest that Sirt6 is a critical anti-aging molecule that regulates various cellular processes associated with aging and protects the heart from developing aging-induced cardiac hypertrophy and fibrosis.

## INTRODUCTION

Age is an important determinant of cardiovascular health [1, 2]. Correspondingly, the prevalence of cardiovascular diseases increases from about 40% in persons of 40-59 years of age, 70-75% in persons of 60-79 years of age, and 79-86% among those aged 80 years or older [3]. As the US population is projected to grow from 314 million in 2017 to 400 million in the year 2030, nearly 1 in 5 individuals will be 65 years or older, implying that with the current trend, cardiovascular disease will be the number one killer of Americans in the near future [3]. Hence, identifying cardiac aging mechanisms in which therapeutic interventions are possible is of great significance.

The non-genetic interventions that have been shown to slow the aging process are calorie restriction and endurance exercise [4]. A plethora of evidence suggests that these interventions work by changing the activity of a group of histone deacetylases called sirtuins [5]. Among the seven sirtuin isoforms encoded by the mammalian genome, Sirt6, a nuclear sirtuin, has been shown to extend lifespan in mice [6]. Correspondingly, Sirt6 deficient mice show a premature aging phenotype with metabolic defects and also have a lifespan of only four weeks [7]. Our previous studies have demonstrated that Sirt6 protects the heart from developing pressure overload hypertrophy and obesity induced diabetic cardiomyopathy, suggesting that Sirt6 may also retard the cardiac aging induced hypertrophy [8, 9].

Several aging theories have been proposed, including the free radical, mitochondrial, gene dysregulation, immunological, telomere, and inflammation theory of aging [10]. Studies on these particular theories have provided valuable insights into the mechanisms of aging. The existence of these many theories is suggestive of a complex and multifactorial etiology in aging. Therefore, identifying a molecule that plays a pivotal role in regulating a variety of the cellular processes involved in aging is of utmost importance.

According to the telomere erosion theory of aging, repetitive telomere sequences at the end of chromosomes shorten with age, which is considered a hallmark of aging [11]. A highly conserved group of proteins called the shelterin complex protects chromosome ends (telomeres) from degradation and inappropriate DNA damage response (DDR), thus preserving chromosome stability and integrity [12]. The activity of the enzyme telomerase maintains telomere length. Each cell division results in the loss of 30-200 nucleotides of the telomere and, thus, acts as a mitotic clock that measures the number of times a cell has divided [12]. Although cardiomyocytes are mostly terminally differentiated cells, they also express functionally significant telomerase levels [13, 14]. Several epidemiological studies have shown an association between short telomere lengths to the development of cardiovascular diseases and their related mortality [15]. A recent study has reported that cardiomyocyte-specific telomere shortening is a distinct signature of heart failure in humans [16]. Sirt6 is associated with telomeres, and depletion of Sirt6 causes telomere dysfunction with end-to-end chromosomal fusions, leading to premature cellular senescence [17]. Further, Sirt6 is required for oxidative damage-induced telomeric movement thus facilitating efficient telomere damage repair [18]. While these studies have suggested that deficiency of Sirt6 is detrimental to telomere maintenance, the role of Sirt6 in maintaining telomeres in aging mice is not yet reported.

Telomere dysfunction can also compromise cardiomyocyte function by impairing mitochondrial biogenesis [19]. Mitochondria play an essential role in regulating cardiovascular pathophysiology and, hence, it is considered to be one of the potential therapeutic targets for treating heart failure [20]. Mitochondrial substrate metabolism is tightly linked to the availability of nicotinamide adenine dinucleotide (NAD^+^) [21]. One of aging's characteristic features is the decline of NAD^+^ levels and reduced mitochondrial function [21]. Hence, elevating NAD^+^ levels is considered as a viable therapeutic strategy to retard the aging process. We and others have shown that the exogenous supplementation of NAD^+^ and its precursors, or the manipulation of critical enzymes within its biosynthetic pathways, is cardioprotective against a variety of stressors [22–25]. The activity of sirtuins is intricately tied to NAD^+^ metabolism because of its dependence on NAD^+^. Several studies have shown that many of the beneficial effects of NAD^+^ or its precursors on cardiomyocytes are mediated through activation of Sirt1 and Sirt3 [22]. However, the role of Sirt6 in the maintenance of cardiac NAD^+^ levels is largely unexplored.

Mitochondrial dysfunction associated with aging is also correlated with increased mitochondrial DNA damage. The circular mitochondrial DNA in mammals encodes 37 genes, including 2 rRNAs, 22 tRNAs, and 13 polypeptides [26]. All 13 polypeptides are components of the oxidative phosphorylation (OXPHOS) system. Because of its proximity to ROS production and lack of histones, mitochondrial DNA is more vulnerable to damage. It has more than a 10-17 fold increased mutation rate compared to nuclear DNA [27]. The mitochondrial theory of aging proposes that ROS-induced oxidative damage in mitochondrial DNA causes mutations, leading to the synthesis of defective polypeptide components of the electron transport chain (ETC). Defective ETC components further increase ROS production, initiating a vicious cycle of mitochondrial DNA mutations and dysfunction [28].

To effectively distribute mitochondrial-gene encoded proteins, mitochondrial DNA is uniformly spread throughout the mitochondrial network. This is achieved by continuous fusion and fission between mitochondria [29]. Defective mitochondrial fusion and fission dynamics are detrimental to mitochondrial DNA (mtDNA). Mice with deficiencies in Mfn1 and Mfn2, mitochondrial outer membrane fusion proteins, develop muscle atrophy associated with mitochondrial dysfunction and severe mtDNA depletion [30]. Similarly, mutations in OPA1, a mitochondrial inner membrane fusion protein, is also associated with mtDNA replication, distribution, and maintenance [31]. Correspondingly, loss of DRP1, a mitochondrial fission protein, causes hyper-fused dysfunctional mitochondria with enlarged mtDNA nucleoids, characterized by mtDNA accumulation [32].

Using cell culture and animal models, this study demonstrates that Sirt6 acts on multiple components of aging mechanisms to retard the cardiac aging process.

## RESULTS

**Figure 1:**
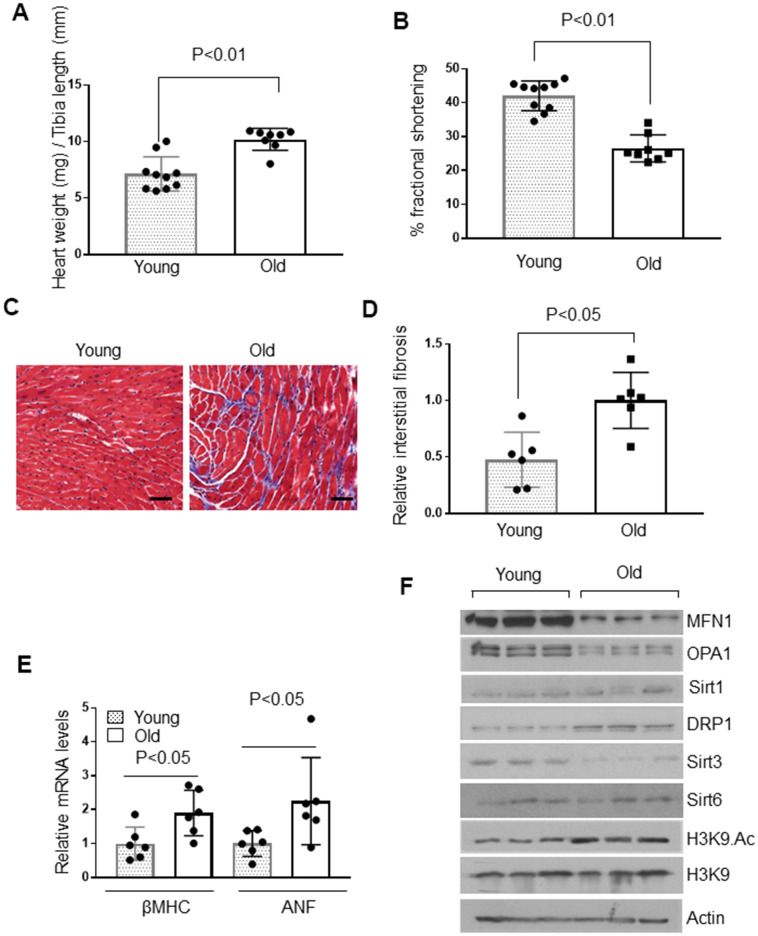
**Aged mice show cardiac hypertrophic response.** (**A**) Heart weight to tibia length (HW/TL) ratio of 4-month-old (Young) and 24-month-old (Old) mice. Values are mean ± SE, n = 8-10. (**B**) Echocardiographic measurements of fractional shortening in young and old mice. Values are mean ± SE, n = 8-10. (**C**) Representative sections of hearts stained with Masson's trichrome to detect fibrosis (blue); scale bars, 20 μm. (**D**) Quantification of cardiac fibrosis in young and old mice. Mean ± SE, n = 6. (**E**) Expression levels of βMHC and ANF mRNA in young and old mice, mean ± SE, n = 6 mice. (**F**) Heart lysates of young and old mice were subjected to immunoblotting using indicated antibodies. Representative blots of three different mice in each group are shown, n = 6. (Quantification of blots is given in Supplementary Figure 3A–3E).

The decline of cardiac functions with age is associated with development of cardiac hypertrophy and fibrosis [33, 34]. Therefore, we examined the hearts of 4-month and 24-month-old mice to determine the extent of cardiac hypertrophy. Old mice showed a significant increase in heart weight to tibia length ratio than younger mice (Figure 1A). Echocardiography of these mice revealed significantly reduced fractional shortening among old mice (Figure 1B). Old mice also showed increased interstitial fibrosis, as measured by Masson’s trichrome staining (Figure 1C, 1D). Additionally, old mice showed increased expression of fetal genes, β Myosin heavy chain (β-MHC) and atrial natriuretic factor (ANF), suggesting that there is well-established aging-induced cardiac hypertrophy in 24-month-old mice, compared to 4-month-old adult mice (Figure 1E). Our previous study showed that Sirt6 is a cardioprotective molecule, and the loss of Sirt6 leads to heart failure [8]. Therefore, we tested Sirt6 levels in young and old mice. We did not find any appreciable differences in Sirt6 protein and mRNA levels between these two groups of mice (Figure 1F and Supplementary Figure 1A, 1B). Hence, we presumed that Sirt6 enzymatic activity could be different in young and old mice. To test this hypothesis, we immunoprecipitated Sirt6 from the hearts of young and old mice, and then we tested Sirt6 activity using a fluorometric assay kit. Interestingly, we did not observe any difference in Sirt6 activity between these two groups of mice (Supplementary Figure 1C).

One of the fundamental observations about the role of sirtuins in aging yeast is that they dissociate from silent mating-type genes and relocate to DNA breaks to facilitate DNA repair, causing old cells to become sterile during DNA damage [35–37]. Therefore, we posited that Sirt6 might be less available at the chromatin to silence genes in senescent cells due to increased cellular stress and DNA damage. At the chromatin, Sirt6 represses gene transcription by deacetylating histone H3 (H3K9) [17]. For that reason, we tested the H3K9 acetylation status in young and old mice. We found that aged mice have increased H3K9 acetylation, suggesting the possible relocalization of Sirt6 to regions other than H3 (Figure 1F and Supplementary Figure 2A).

Sirt6 interacts with the stress-responsive transcription factor NF-κB and suppresses NF-κB target gene expression by deacetylating H3K9 [38]. Hence, we tested the mRNA expression of NF-κB target genes, IL-6 and Cdkn1a [39, 40]. Consistent with increased H3K9 acetylation levels, IL-6 and Cdkn1a (p21) mRNA expression were found to be upregulated in old mice compared to younger animals, suggesting decreased localization of Sirt6 to the chromatin (Supplementary Figure 2B, 2C).

We also measured the expression levels of other sirtuins in the heart of young and old mice. We did not find any difference in Sirt1, but Sirt3 levels were significantly reduced in aged mice (Figure 1F and Supplementary Figure 3A, 3B). This was associated with reduced levels of OPA1 and MFN1 (proteins involved in mitochondrial fusion) and increased expression of DRP1 (a protein involved in mitochondrial fission) in aged mice, compared to younger animals (Figure 1F and Supplementary Figure 3C–3E). Thus, these data indicated that, compared to young mice, aged animals have well-established cardiac hypertrophy associated with reduced Sirt6 availability at the chromatin and dysregulation of mitochondrial Sirt3 and fusion-fission dynamics.

**Figure 2:**
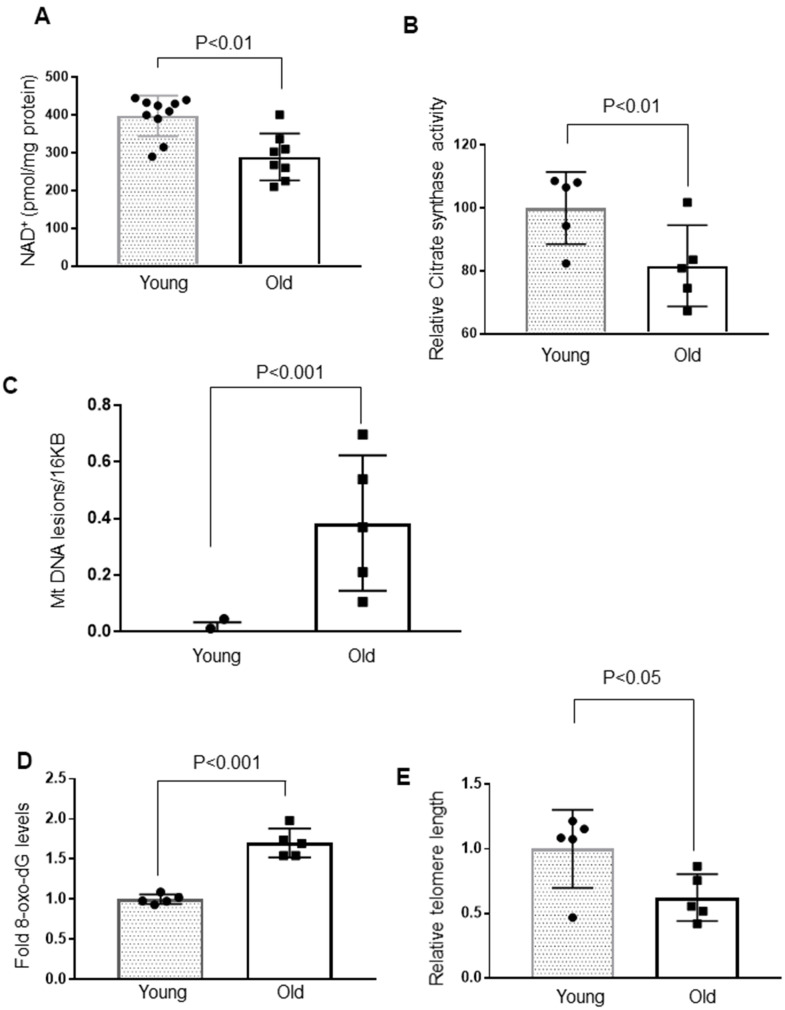
**Cardiac aging is associated with decreased mitochondrial function and increased mtDNA and telomere damage.** (**A**) Quantification of NAD^+^ in the heart lysate of Young and Old mice. Values are mean ± SE, n = 8-10. (**B**) Relative mitochondrial citrate synthase activity in the heart of Young and Old mice. Values are mean ± SE, n = 5. (**C**) Relative mitochondrial DNA lesions in the heart of Young and Old mice. (**D**) 8-Oxo-dG content in the DNA of the whole heart of Young and Old mice. All values are mean ± SE, n = 5. (**E**) Relative telomere length of Young and Old mice. Values are mean ± SE, n = 5.

Nicotinamide adenine dinucleotide (NAD^+^) is fundamental to cellular energy metabolism, and depletion of NAD^+^ is one of the hallmarks of aging and age-related diseases [21]. Therefore, we compared the intracellular NAD^+^ levels in young and old mice, and found that aging caused depletion of cardiac NAD^+^ levels by ~40% (Figure 2A). Due to the decreased Sirt3 and NAD^+^ levels, we speculated that the activity of citrate synthase, a substrate of Sirt3, could be affected. Citrate synthase is a mitochondrial enzyme involved in the first step of the TCA cycle. It catalyzes acetate and oxaloacetate condensation to form citrate and, thus, is a key marker for mitochondrial metabolic function [41]. As expected, citrate synthase activity was significantly reduced in aged mice (Figure 2B). The compromised function of mitochondria is also associated with increased mitochondrial DNA lesions. In agreement with this, we found significantly increased mitochondrial DNA damage in older mice (Figure 2C). Earlier studies have documented an age-associated increase in oxidative DNA damage, which can be measured by determining the accumulation of 8-oxo-dG adducts (7-8-dihydro-8-oxo-2 deoxyguanosine) [42, 43]. Aged mice hearts showed a significant accumulation of 8-oxo-dG adducts compared to younger mice (Figure 2D). We also found increased telomere loss in aged mice, yet another hallmark of aging (Figure 2E). Together, these results suggest that cardiac aging is associated with altered functions of Sirt3, Sirt6 and loss of mitochondrial DNA integrity.

**Figure 3:**
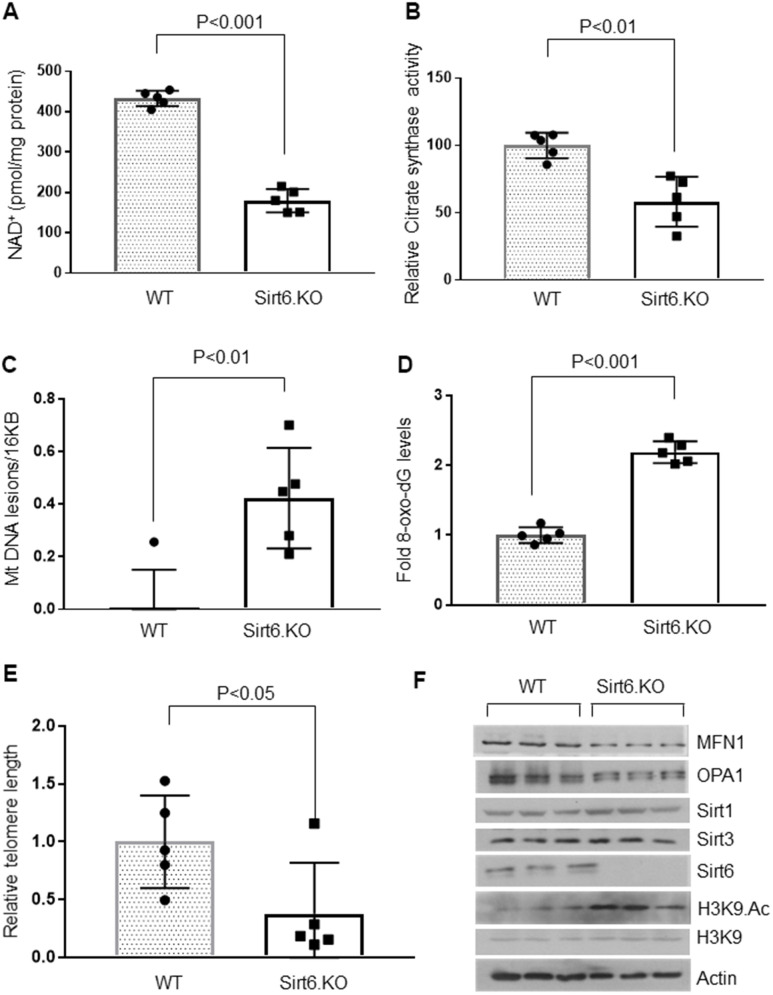
**Sirt6.KO mice display cardiac aging phenotype.** (**A**) Quantification of NAD^+^ contents in the heart lysate of wild type and Sirt6.KO mice. Values are mean ± SE, n = 5. (**B**) Relative Mitochondrial citrate synthase activity in the heart of Wild type and Sirt6.KO mice. Values are mean ± SE, n = 5. (**C**) Relative mitochondrial DNA lesions in the heart of Wild type and Sirt6.KO mice. Values are mean ± SE, n = 5. (**D**) 8-Oxo-dG content in the DNA of the whole heart of Wild type and Sirt6.KO mice. Values are mean ± SE, n = 5. (**E**) Relative telomere length of Wild type and Sirt6.KO mice. Values are mean ± SE, n = 5. (**F**) Heart lysates of Wild type and Sirt6.KO mice were subjected to immunoblotting using indicated antibodies. Representative blots of three different mice in each group are shown, n = 6. (Quantification of blots is given in Supplementary Figure 4A–4E).

To test whether Sirt6 deficiency contributed to cardiac aging, we analyzed the hearts of whole-body Sirt6 knockout mice. Like aged mice, four weeks old, Sirt6 deficient mice showed decreased cardiac NAD^+^ levels and decreased citrate synthase activity compared to their wild-type littermates (Figure 3A, 3B). Hearts of Sirt6.KO mice also showed increased mitochondrial DNA lesions and increased accumulation of 8-oxo-dG adducts (Figure 3C, 3D). Sirt6 deficient hearts also showed significantly shortened telomeres (Figure 3E). We also analyzed Sirt6.KO hearts for proteins associated with perturbations in mitochondrial dynamics. The western blot analysis showed increased H3K9 acetylation (Figure 3F and Supplementary Figure 4C) and reduced levels of OPA1 and MFN1 (Figure 3F and Supplementary Figure 4A, 4B) in Sirt6 deficient hearts, compared to the wild-type controls. However, we did not find any difference in Sirt1 and Sirt3 levels between wild-type and Sirt6.KO mice (Figure 3F and Supplementary Figure 4D, 4E). These results suggest that Sirt6 deficient heart shows characteristics of aging.

**Figure 4:**
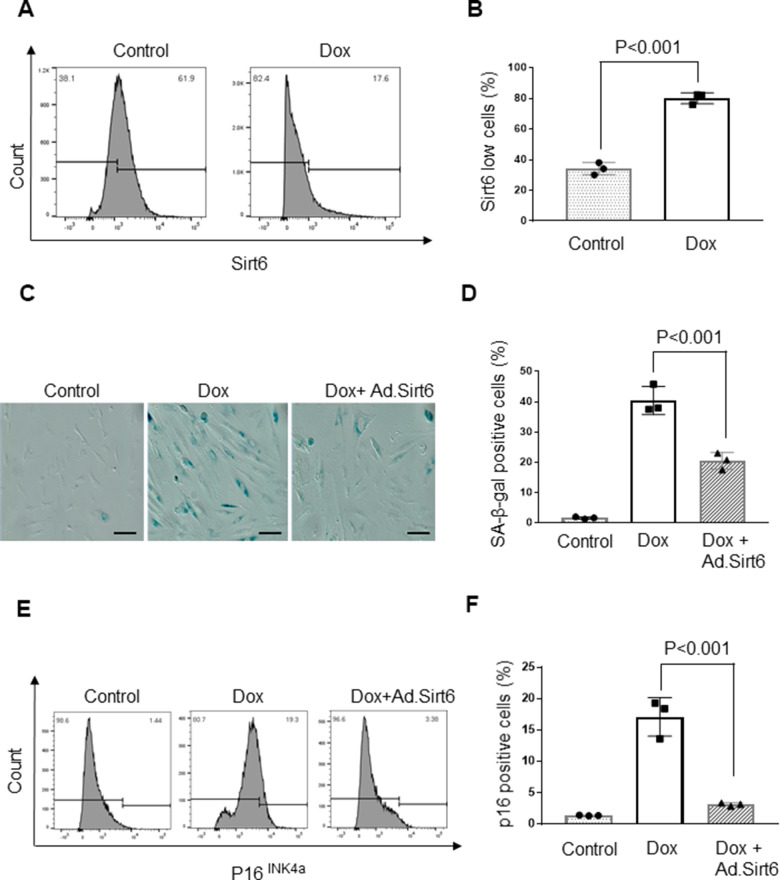
**Sirt6 overexpression protects cardiomyocytes from senescence.** (**A**) Flow cytometric analysis of senescence induced H9c2 cardiomyocytes. The Gated region indicates percentages of cells expressing low levels of SIRT6. Values are the average of three independent experiments, Mean ± SE. (**B**) Quantification of SIRT6 in samples shown in (**A**). (**C**) Representative images of β-galactosidase staining of control and senescence induced cardiomyocytes transduced with empty or SIRT6 adenovirus scale bars, 100 μm. (**D**) Quantification of senescent cells in (**C**). Values are average of three independent experiments, Mean ± SE. (**E**) Flow cytometric analysis of control and senescence induced cardiomyocytes transduced with empty or SIRT6 adenovirus. The Gated region indicates the percentages of cells expressing p16. Values are the average of three independent experiments, Mean ± SE. (**F**) Quantification of p16 levels in samples shown in (**E**).

Since Sirt6 deficiency mimics the aging phenotype, we examined the effect of Sirt6 overexpression in an *in vitro* model of cardiomyocyte senescence. To induce senescence *in vitro*, H9c2 cardiomyocytes were treated with 0.5 μM doxorubicin (Dox). Following 24 hours of treatment, Dox was removed, and cells were maintained in 10% FBS containing DMEM for 10 days. Cells were harvested and analyzed by western blotting or subjected to FACS analysis. We found that Dox treated cells had 50% reduced Sirt6 levels than controls, supporting our hypothesis that senescence negatively affects the Sirt6 level (Figure 4A, 4B). Having found that Sirt6 levels were reduced following Dox-induced senescence, we next investigated whether over-expression of Sirt6 can prevent Dox-induced β-galactosidase activity, a marker of cellular senescence. β-galactosidase is a lysosomal enzyme that can be detected at an optimal pH 4, but if cells express high levels of beta-galactosidase, it can be detected even in less favorable pH conditions (pH 6). We found that β-galactosidase was expressed at high levels in Dox treated cells at pH 6, whereas adenovirus mediated overexpression of Sirt6 blocked the induction of β-galactosidase (Figure 4C, 4D). Senescent cells often express p16^INK4a^, a cyclin-dependent kinase inhibitor, and p53, a cell cycle inhibitor that is considered an important biomarker of aging. Therefore, we also measured p16^INK4a^ levels in Dox-induced cells transduced with Ad.Sirt6 (5 MOI) or control virus. Both vehicle and Dox treated cells were infected with 5 MOI of empty adenovirus. While expression of p16^INK4a^ was significantly increased in Dox-induced senescent cells, Sirt6 overexpression dampened p16^INK4a^ expression in cells subjected to senescence (Figure 4E, 4F). These results demonstrate the anti-senescence effects of Sirt6 in cardiomyocytes.

**Figure 5:**
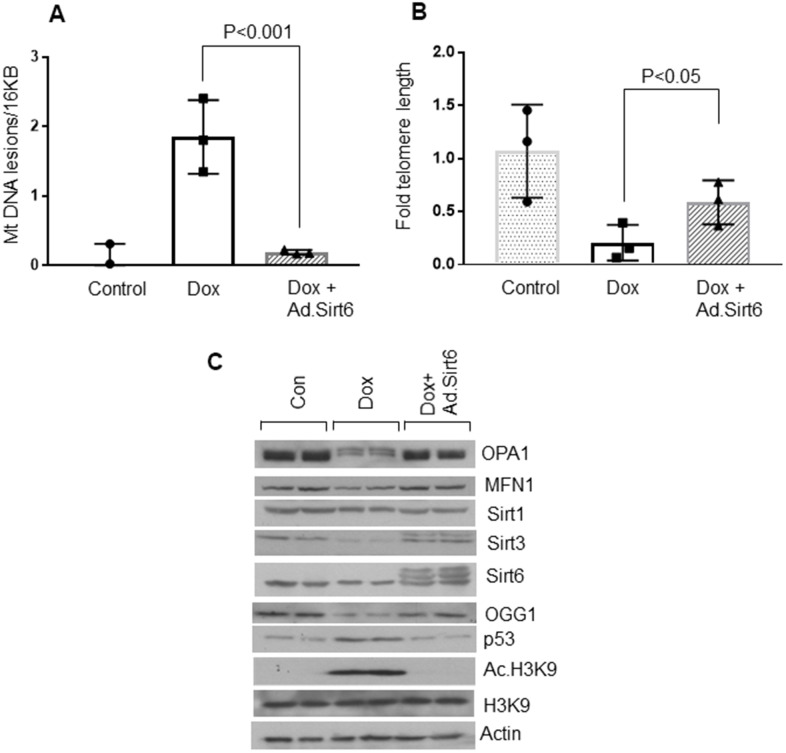
**Sirt6 overexpression protects cardiomyocytes from mitochondrial and telomere DNA damage.** (**A**) Relative mitochondrial DNA lesions in the control and senescence induced cardiomyocytes transduced with or without SIRT6. Values are the average of three independent experiments, Mean ± SE. (**B**) Relative telomere length in the control and senescence induced cardiomyocytes transduced with empty or SIRT6 adenovirus. Values are the average of three independent experiments. Mean ± SE. (**C**) H9c2 cardiomyocytes were treated with doxorubicin in the presence or absence of SIRT6 to induce senescence. Cell lysate was prepared and analyzed by immunoblotting using indicated antibodies. Representative blot of three independent experiments showing two different samples in each group (quantification of blots is given in Supplementary Figure 5A–5G).

Telomere dysfunction has been shown to induce mitochondrial dysfunction in the heart [44]. To further investigate anti-senescence characteristics of Sirt6, we measured mitochondrial DNA lesions and telomere length in H9c2 cardiomyocytes subjected to undergo senescence, with or without Sirt6 over-expression. Consistent with our previous observations, Sirt6 over-expressed cardiomyocytes showed reduced mitochondrial DNA damage, and longer telomere length than cells not receiving Sirt6 overexpression, confirming that Sirt6 can protect cells from undergoing senescence (Figure 5A, 5B). We also assessed mitochondrial health by determining mitochondrial protein levels in the same groups of cells. Consistent with the above results, senescent cells showed reduced levels of mitochondrial proteins, OPA1, MFN1, OGG1, and Sirt3, and increased levels of p53 and acetylated H3K9. However, Sirt1 levels remain unchanged. Correspondingly, Sirt6 overexpressed cells maintained these protein levels similar to that of controls (Figure 5C and Supplementary Figure 5A–5G). These results strongly indicate that Sirt6 could block the senescence of cardiomyocytes.

**Figure 6:**
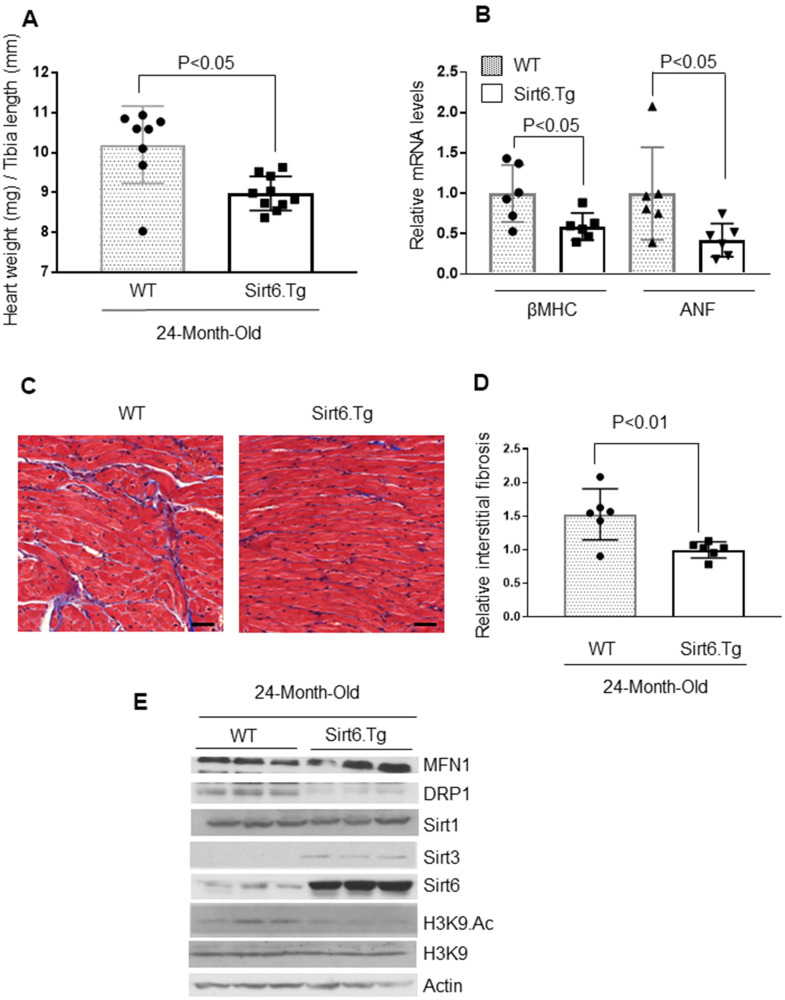
**Sirt6.Tg mice are protected from aging induced cardiac hypertrophy.** (**A**) Heart weight to tibia length (HW/TL) ratio of 24-month-old Wild type and 24-month-old Sirt6 transgenic (Sirt6.Tg) mice. Values are mean ± SE, n = 8-10. (**B**) Expression levels of βMHC and ANF mRNA levels in Wild type and Sirt6.Tg mice, mean ± SE, n=6 mice. (**C**) Representative sections of hearts stained with Masson's trichrome to detect fibrosis (blue); scale bars, 20 μm. (**D**) Quantification of cardiac fibrosis in Wild type and Sirt6.Tg mice. Mean ± SE, n = 6. (**E**) Heart lysates of Wild type and Sirt6.Tg mice were subjected to immunoblotting using indicated antibodies. Representative blots of three different mice in each group are shown, n = 6. (Quantification of blots is given in Supplementary Figure 6A–6F).

To evaluate the anti-aging effects of Sirt6 *in vivo*, we generated whole body Sirt6 over-expressing transgenic mice, and maintained them for 24-months, along with their non-transgenic wild-type littermates [9]. Sirt6.Tg mice showed reduced cardiac hypertrophy, as measured by a decreased HW/TL ratio and mRNA levels for βMHC and ANF, compared to non-transgenic wild-type controls (Figure 6A, 6B). Additionally, WT mice also showed increased fibrosis levels than Sirt6.Tg mice (Figure 6C, 6D). Further, mitochondrial protein analysis showed increased expression of MFN1 and decreased expression of mitochondrial fission protein DRP1 in Sirt6.Tg mice hearts, compared to same-age wild-type controls. Sirt6.Tg hearts also showed increased levels of Sirt3 and decreased levels of acetylated H3K9. No change in Sirt1 levels was observed (Figure 6E and Supplementary Figure 6A–6F). Thus, these data indicate that Sirt6 overexpression could block the development of pathologic changes associated with cardiac aging.

**Figure 7:**
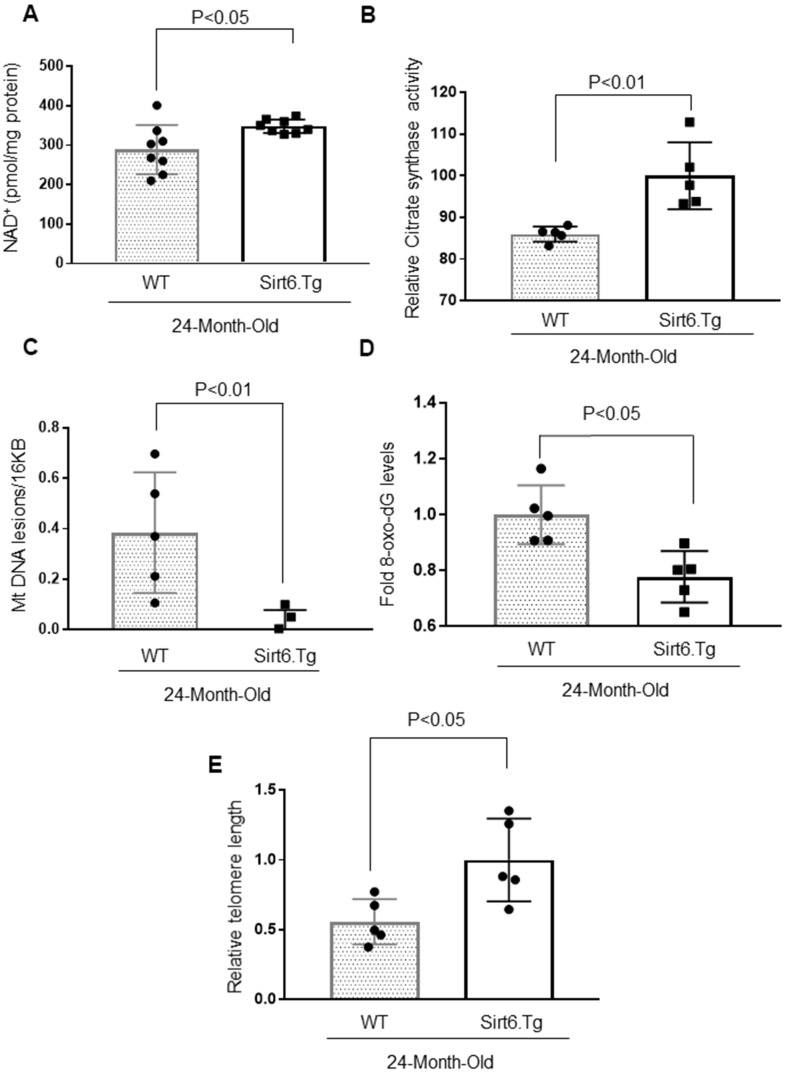
**Sirt6.Tg mice display reduced cardiac aging phenotype.** (**A**) Quantification of NAD^+^ contents in the heart lysate of 24-month-old WT and 24-month-old Sirt6 transgenic (Sirt6.Tg) mice. Values are mean ± SE, n = 8. (**B**) Relative Mitochondrial citrate synthase activity in the heart of Wild type and Sirt6.Tg mice. Values are mean ± SE, n = 5. (**C**) Relative mitochondrial lesions in the heart of Wild type and Sirt6.Tg mice. Values are mean ± SE, n = 5. (**D**) 8-Oxo-dG content in the DNA of the whole heart of Wild type and Sirt6.Tg mice. Values are mean ± SE, n = 5. (**E**) Relative telomere length of Wild type and Sirt6.Tg mice. Values are mean ± SE, n = 5.

To further confirm these results, we measured cardiac NAD^+^ levels and citrate synthase activity in the hearts of 24-months-old wild-type and Sirt6.Tg mice. Sirt6.Tg mice showed increased NAD^+^ levels and citrate synthase activity (Figure 7A, 7B). We also determined the mitochondrial DNA lesions and 8-oxo-dG adducts in these mice and found that both parameters were significantly reduced in Sirt6.Tg mice (Figure 7C, 7D). Sirt6.Tg mice also had longer telomeres in the hearts than their wild type littermates (Figure 7E). These findings collectively suggest that Sirt6 is an anti-senescence molecule and protects the heart from developing aging-associated cardiac hypertrophy.

## DISCUSSION

This study was designed to investigate the effect of Sirt6 on cardiac aging. We demonstrated that 24-months aged mice developed cardiac dysfunction associated with cardiac hypertrophy and fibrosis. These mice also showed reduced NAD^+^ levels, increased mitochondrial DNA damage, shortened telomere length, and increased accumulation of 8-oxo-dG adducts, which are considered hallmarks of aging. Further, aged mice showed perturbed levels of proteins involved in mitochondrial dynamics. A similar phenotype was observed in Sirt6 deficient mice. However, Sirt6 overexpression blocked cardiomyocyte senescence *in vitro* and cardiac hypertrophic response *in vivo*. Sirt6.Tg mice also had reduced cardiac fibrosis and decreased expression of aging markers. These studies reveal that Sirt6 protects the heart from developing aging-associated pathologies.

In humans, age is a fundamental predictor of cardiovascular risk, even after adjusting for other traditional risk factors, including gender, smoking, HDL/LDL-cholesterol, and blood pressure [1]. To confirm the presence of age-related cardiac pathology, we first compared the hearts of young mice with old mice. 24 months old mice were considered aged because previous studies have established cardiac pathologies at this age in mice [45]. As expected, we found that old mice, as well as Sirt6.KO mice have increased cardiac pathology and expression of associated aging biomarkers, whereas Sirt6.Tg mice were protected from developing aging-associated cardiac changes.

Interestingly, we found reduced levels of Sirt3 in the old mice hearts, but not Sirt6. One of the well-established substrates of Sirt6 is histone H3K9, whose deacetylation leads to transcriptional silencing [46]. In this study, we found increased acetylation of H3K9 in old mice hearts. We speculated that this could be because of decreased activity of Sirt6 at H3 of chromatin. Surprisingly, the activity assay we performed using Sirt6 immunoprecipitated from young, and aged mice showed no difference in activity. One caveat of this activity assay is that it was performed with an excess of NAD+ (800μM), which contrasts to the lower NAD+ concentrations observed in the aged mice. Hence we warrant caution while interpreting this result. Another reason for increased H3K9 acetylation could be because of the altered localization of the Sirt6 at the chromatin. We have observed increased DNA damage in aged mice as evidenced by increased 8-oxo-dG adducts. Hence we speculate that in aged mice, Sirt6 may be involved in DNA damage repair rather than localizing at the histones to silence the expression of senescence genes [47]. In agreement with this, we found increased mRNA expression of IL-6 and p21, two senescence-associated genes that are regulated by the stress-responsive transcription factor NF-κB. Several studies have convincingly shown that Sirt6 negatively regulates NF-κB responsive genes through H3K9 deacetylation at their promoters [38, 48]. Our results suggest that increased H3K9 acetylation in aged mice hearts could be because of decreased NAD+ levels, altered localization of Sirt6 or due to a combination of both. Previous studies have also found increased H3K9 acetylation during aging in D. melanogaster [49]. To the best of our knowledge, no previous report is available regarding the acetylation status of H3K9 in aged mouse hearts. Additionally, the reduced level of Sirt3 in the context of altered Sirt6 localization can be explained based on our recent publication, where we have shown that Sirt6 suppresses the expression of Keap1, a negative regulator of Nrf2, and thereby abolishing Keap1-Nrf2 binding in the cytoplasm. Consequently, free Nrf2 translocates to the nucleus and initiates the transcription of Sirt3 and other antioxidant genes [9].

We also observed dysregulated levels of mitochondrial fission and fusion proteins in old mice and Sirt6.KO mice hearts. However, in contrast to aged mice, we did not see any change in Sirt3 level in Sirt6.KO mice, suggesting that there are additional mechanisms involved in the regulation of Sirt3. Mitochondria are dynamic organelles, and frequent mitochondrial fission and fusion mediate the elimination of impaired mitochondrial components. Defects in mitochondrial dynamics affect bioenergetic levels, leading to mitochondrial dysfunction, apoptosis, and aging. The dynamic nature of mitochondria is necessary for a cell to respond to its ever-changing physiological conditions [50]. In general, mitochondrial fusion is beneficial in metabolically active cells, whereas in quiescent cells, mitochondrial fission is favored [51, 52]. Because the heart is the most metabolically demanding organ in the body, mitochondria occupy one-third of cell-volume in cardiomyocytes, making them the cell-type with the highest mitochondria content [53]. Impaired mitochondrial fusion by deleting MFN1/MFN2 or OPA1 leads to cardiac dysfunction [54–56]. A similar effect was observed in a mouse model of cardiac arrest. The inhibition of mitochondrial fission protein, dynamin-related protein 1 (DRP1), improved the time to return spontaneous circulation and cardiac hemodynamics, resulting in improved animal survival in the model of cardiac arrest [57]. Our observation that Sirt6.Tg old mice exhibit normal levels of mitochondrial fusion-fission proteins suggests the important role of Sirt6 in regulating mitochondrial health.

Changes in mitochondrial bioenergetics can lead to perturbations in cellular NAD+ levels [58]. NAD+ is a coenzyme needed for oxidoreductase reactions in energy metabolism. All the major energy-generating pathways, including glycolysis, fatty acid β-oxidation and Krebs cycle, reduce NAD+ to NADH. The latter acts as a major electron donor to the electron transport chain in mitochondria to synthesize ATP [58]. NAD+ also serves as a precursor for NADP. The reduced form of NADP (NADPH) plays a vital role in the negative regulation of ROS production. Recently, many studies have shown that aging is associated with a decline in NAD+ levels resulting from metabolism dysregulation [59]. Aged mouse or human tissues have only half the NAD+ levels than normal healthy tissues [60, 61]. NAD+ has a pleiotropic effect in the cell, capable of inducing a multitude of cellular effects by impacting energy metabolism, protein modifications such as ADP ribosylation, deacetylation, and cellular signaling [62].

Similarly, the importance of Sirt6 in regulating NAD^+^ levels is quickly emerging. It was recently shown that Sirt6 could deacetylate Nampt to increase its activity, thereby increasing the synthesis of NAD^+^ [63]. SIRT6^+/−^ mice showed significantly decreased levels of NAD^+^ in the liver and pancreas [63]. These data are in line with our observation that old mice and Sirt6 deficient mice showed decreased cardiac NAD^+^ levels compared to their controls. Correspondingly, in the present study, Sirt6.Tg mice showed increased NAD^+^ levels when compared to their wild-type littermates. For the first time, our data show a direct link between NAD^+^ levels and altered Sirt6 activity during cardiac aging.

On a similar note, decreased mitochondrial function with aging is observed in many different mammalian species, including humans, where it is correlated with increased mitochondrial DNA mutations [64]. This is in accordance with the mitochondrial theory of cellular aging. According to this theory, mitochondria have limited ability to repair mutations, causing accumulation of mutations over time, resulting in increased oxidative stress, compromising mitochondrial function [64]. The effects of mitochondrial mutations are more pronounced in post-mitotic organs like the heart [65, 66]. Our findings show that Sirt6 is involved in protecting mitochondria from accumulating mitochondrial DNA damage upon aging. Details of the mechanism through which Sirt6 protects cardiac mitochondrial DNA warrants further investigation.

Increased prevalence of mitochondrial DNA mutations in the aging heart is also reflected in the decline of mitochondrial function. Mitochondrial citrate synthase (mCS) is the first rate-limiting enzyme of the tricarboxylic acid (TCA) cycle. It catalyzes the condensation of acetate and oxaloacetate to form citrate, and hence a key marker for mitochondrial function. Old mice showed decreased citrate synthase activity, but this decline in activity was not observed in Sirt6.Tg mice. Correspondingly, Sirt6.Tg mice also showed reduced accumulation of oxidative DNA lesion, 8-oxo-deoxyguanosine. Together, these results indicate that Sirt6 can protect cardiomyocytes from oxidative damage and maintain mitochondrial function, consequently retarding the aging process.

Another interesting observation that we have made in the present study is the role of Sirt6 in protecting the cardiomyocytes telomere DNA. Telomeres consist of tandem repeats of the TTAGGG DNA sequence bound by a six-protein complex known as shelterin [12, 67]. The enzyme that helps in adding the TTAGGG sequence is called telomerase [68]. Telomerase has two subunits, a reverse transcriptase catalytic subunit (Tert), and an associated RNA component (Terc), which is used as a template for the synthesis of TTAGGG repeats [69]. In the Tert −/− and Terc −/− mice, telomere dysfunction induces mitochondrial defects, leading to cardiac aging and heart failure [44, 70]. Correspondingly, mice with hyper-long telomeres show less metabolic aging and extended lifespan [69]. Even though the effect of shortened telomeres in cardiac aging is controversial, several studies have reported cardiomyocyte telomere shortening in heart diseases [16, 71]. Sharifi-Sanjani et al have shown that myocardial telomere length attrition occurs at a rate of 20 bp per year [16]. Telomere length analysis performed in biopsies of diseased heart tissues show an unequivocal association between shortened telomeres and cardiac diseases [14, 16, 71, 72]. Further, in line with our observation, cardiomyocyte telomere shortening in hypertrophic cardiomyopathy is associated with extensive DNA damage, suggesting that shortened telomeres may be causative of cardiac hypertrophy [16].

Our results *in vitro* and *in vivo* suggest that loss of Sirt6 induces telomere attrition in cardiomyocytes, whereas overexpression of Sirt6 prevents telomere shortening. These findings are supported by previous studies, where Sirt6 was shown to be required for proper maintenance of telomere length and function [17]. Deacetylation of H3K9 at telomeric chromatin enables stable association of WRN, the factor that is mutated in Werner syndrome, a human premature aging syndrome [17]. Further, reduced Sirt6 levels after transverse aortic constriction in mice were found to be associated with downregulation of telomerase reverse transcriptase (Tert), and telomeric repeat binding factor (TRF-1) [73]. Together, these studies reveal an essential role of Sirt6 in the protection of cardiomyocytes from developing senescence.

As discussed, aging is a multifaceted phenomenon. Many different proteins and enzymes work in concert to maintain cellular homeostasis. Hence, implementing a more generalized strategy rather than correction of a specific defect is critical in managing aging-associated diseases. Our data suggest that Sirt6 is a pivotal molecule that can regulate various aging pathways and thus could be a potential therapeutic target to retard cardiac aging.

## MATERIALS AND METHODS

### Citrate synthase activity assay

Citrate Synthase activity was measured using a citrate synthase activity kit from BioVison Inc, according to the manufacturer's protocol.

### Mice

Sirt6 WT and KO mice (4 weeks old) (Jackson Laboratory, stock number 006050) were on 129svJ background. Sirt6.Tg transgenic mice were generated and characterized as described earlier [9]. Sirt6.Tg, young (4-month-old), and old wild (24-month-old) type mice are of C57Bl6 background. All animal protocols were reviewed and approved by the University of Chicago Institutional Animal Care and Use Committee. All methods were performed in accordance with the relevant guidelines and regulations of the Biosafety Committee of the University of Chicago.

### Cell culture

To induce senescence *in vitro*, H9c2 cardiomyocytes were treated with 0.5 μM doxorubicin. Following 24 hours of treatment, doxorubicin was removed, and cells were maintained in Dulbecco's modified Eagle's medium (DMEM; Invitrogen) supplemented with 1% penicillin-streptomycin and 10% fetal bovine serum (complete growth medium) for 10 days. Sirt6 and empty adenovirus were purchased from Vector Biolabs (Malvern, PA, USA) and were used at an MOI of 5.

### Antibodies and immunoblotting

The antibodies and conjugates used in this study were anti-Mitofusin 1 (MFN1) (MilliporeSigma), anti-SIRT3, anti-SIRT6, anti-Acetyl-Histone H3 Lys9 (Cell Signaling Technology), anti-dynamin-related protein 1 (DRP1), anti-optic atrophy 1 (OPA1) (BD Biosciences), p16INK4A (Thermo Fisher Scientific) anti-tubulin, anti-actin (Santa Cruz Biotechnology), All anti-rabbit, anti-mouse, and anti-goat horseradish peroxidase-conjugated secondary antibodies for western blots were from Santa Cruz Biotechnology. Cells or heart ventricular tissue lysates were prepared in the RIPA buffer 50 mM Tris.HCl (pH 7.5), 150mM NaCL, 1% NP-40, 0.5% Sodium deoxycholate, 0.1% SDS, 1mM DTT, Sigma protease inhibitors and Phosphatase inhibitors. Typically, 20–50 μg of protein lysates were used for immunoblots.

### Measurement of mouse heart function

Chest hair of mice were removed with a topical depilatory agent, and transthoracic echocardiography was performed under inhaled isoflurane (~1%) for anesthesia, delivered via a nose cone. Limb leads were attached for electrocardiogram gating, and the animals were imaged in the left lateral decubitus position with a VisualSonics Vevo 770 machine, using a 30 MHz high-frequency transducer. Body temperature was maintained using a heated imaging platform and warming lamps. Two-dimensional images were recorded in parasternal long- and short-axis projections, with guided M-mode recordings at the midventricular level in both views. LV (left ventricle) cavity size and wall thickness were measured in at least three beats from each projection and averaged. LV fractional shortening ([LVIDd—LVIDs]/LVIDd) was calculated from the M-mode measurements.

### Histology

Heart tissue from mice was fixed in neutral formalin, and sections of the tissues were processed and stained with Masson's trichrome stain to detect fibrosis. Imaging of stained sections was done using Pannoramic Viewer software (3dhistech, Budapest, Hungary), and quantitation was done using ImageJ (National Institutes of Health, Bethesda, MD, USA).

### Mitochondrial DNA damage assay

Total DNA was isolated using Qiagen Genomic-tip 20/G and Qiagen DNA Buffer Set (Qiagen, Gaithersburg, MD) per the manufacturer's instruction. Eluted DNA was incubated with isopropanol overnight at -8° C and centrifuged 12,000g for 60 min. DNA was washed with 70% ethanol and resuspended in Tris.EDTA (TE) buffer. PCR was performed using Ex-taq (Clonetech, Mountain View, CA). Primer sequences for long PCR are: forward 5'cccagctactaccatcattcaagtag3' and reverse, 5'gagagattttatgggtgtaatgcggtg3'. Short PCR was performed using forward primer sequence 5'GCAAATCCATATTCATCCTTCTCAAC3` and the reverse primer sequence the same as long PCR. Resultant PCR products were quantified using Pico-green (Life Technologies). Values obtained from the long fragments were normalized using values from short fragment. The lesion frequency per amplicon was then calculated as *λ* = −ln(*A*D/*A*O), where *A*D/*A*O is the ratio of amplification of the treated samples (*A*D) to the amplification of the control samples (*A*O).

### NAD^+^ and 8-Oxo-dG levels estimation

NAD^+^ levels in the heart were estimated using the NAD/NADH quantitation kit from BioVision Inc, and 8-Oxo-dG levels were estimated using an 8-Oxo-dG ELISA kit from Trevigen, Inc as per manufacturers’ instructions.

### Real-time PCR analysis for mRNA levels

Total RNA was isolated from mouse hearts by using Trizol Reagent (Invitrogen). The residual genomic DNA was digested by incubating the RNA preparation with 0.5 units of RNase-free DNase-1 per microgram of RNA in 1 × reaction buffer for 15 min at room temperature, followed by heat inactivation at 90° C for 5 min. one microgram of DNase-treated RNA were reverse transcribed by RevertAid First Strand cDNA Synthesis Kit (Thermo Fisher Scientific). The resultant cDNA was diluted 10-fold before PCR amplification. A reverse transcriptase minus reaction served as a negative control. The mRNA levels were measured by SYBR green real-time PCR. Primer sequences, ANF forward 5'TCGTCTTGGCCTTTTGGCT3' and reverse 5'TCCAGGTGGTCTAGCAGGTTCT3'. β-MHC, forward 5′AAGGGCCTGAATGAGGAGTA3′ and reverse 5′AAAGGCTCCAGGTCTGAGG3'. Sirt6 forward CCAAATCGTCAGGTCAGGGA and reverse 5′ CAGAGTGGGGTACAGGGATG. IL-6 forward 5′-TGAGAAAAGAGTTGTGCAATGG-3′ and reverse 5′-GGTACTCCAGAAGACCAGAGG-3′. P21 forward 5′-GCAGATCCACAGCGATATCCA-3′ and reverse 5′-AACAGGTCGGACATCACCAG-3′. RPL32 forward 5'ACAACAGGGTGCGGAGAAGATT3' and reverse 5'GTGACTCTGATGGCCAGCTGT 3'. For data analysis, the ΔΔCT (threshold cycle) method was employed [74].

### FACS analysis

Cells were harvested from tissue culture plates and centrifuged at 1,000 rpm for 5 min at 4° C. The supernatant was removed, and cells were washed twice with cold PBS-2%FBS (staining buffer). Intracellular staining for SIRT6 and P16 was performed using an intracellular cytokine staining kit (BD Pharmingen™). Cells were analyzed by flow cytometry using a FacScan analyzer (Becton-Dickinson, San Jose, CA). Results were processed using FlowJo software.

### Telomere length analysis

We used a quantitative PCR method to measure average telomere length ratios, as described previously [75]. In brief, triplicate DNA samples were used for telomeres and their controls, ribosomal protein L30 (Rpl30) for rat, and acidic ribosomal phosphoprotein P0 (36B4) for the mouse. Each reaction included 12.5 μl of Syber Green PCR master mix, 200 nM of each primer, and 10 ng of genomic DNA. The following conditions were used for the PCR reaction. 95° C for 10 minutes, followed by 39 repeats of 95° C for 15 seconds and 60° C for 1 minute, followed by a dissociation stage to monitor amplification specificity. The relative TL was calculated according to the 2^-ΔΔCt^ method. The nucleotide sequences of the PCR primers used were as follows: (written 5’3’):Tel1:CGGTTTGTTTGGGTTTGGGTTTGGGTTTGGGTTTGGGTT;Tel2:GGCTTGCCTTACCCTTACCCTTACCCTTACCCTTACCCT; Rpl30 F:CAGACGCCAAGATGGCCGGG; Rpl30 R:GCTCGGCTTCTGCTTCCGCT;36B4F:ACTGGTCTAGGACCCGAGAAG;36B4R:TCAATGGTGCCTCTGGAGATT.

### Statistical analysis

Statistical differences among groups were determined with either Student's *t*-test (for two groups) or one-way analysis of variance (ANOVA). *P* values less than 0.05 were considered significant.

## Supplementary Material

Supplementary Figures
