# Supplementary material for: The nuclear sirtuin SIRT6 protects the heart from developing aging-associated myocyte senescence and cardiac hypertrophy
Source: Aging (Albany NY). 2021 May 2;13(9):12334–58. doi: 10.18632/aging.203027 (PMC8148452; doi:10.18632/aging.203027)
Supplement: Supplementary Figures [file aging-13-203027-s001.pdf]

SUPPLEMENTARY FIGURES

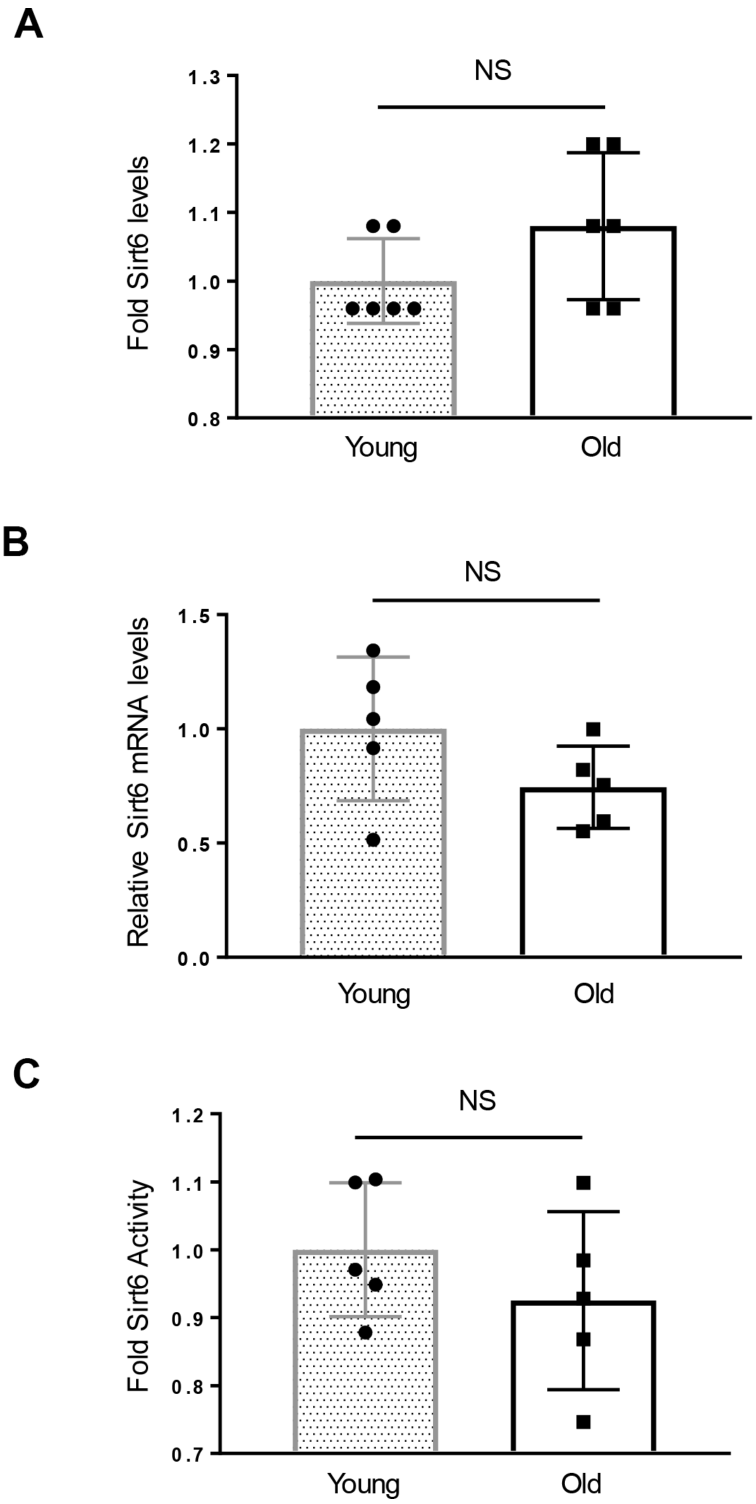

Supplementary Figure 1. SIRT6 levels and enzymatic activity are not affected by aging: (**A**) Relative Sirt6 protein and (**B**) mRNA levels and (**C**) fold Sirt6 activity in the heart of young and old mice. Values are mean ± SE, n = 5, P=NS (Not significant).

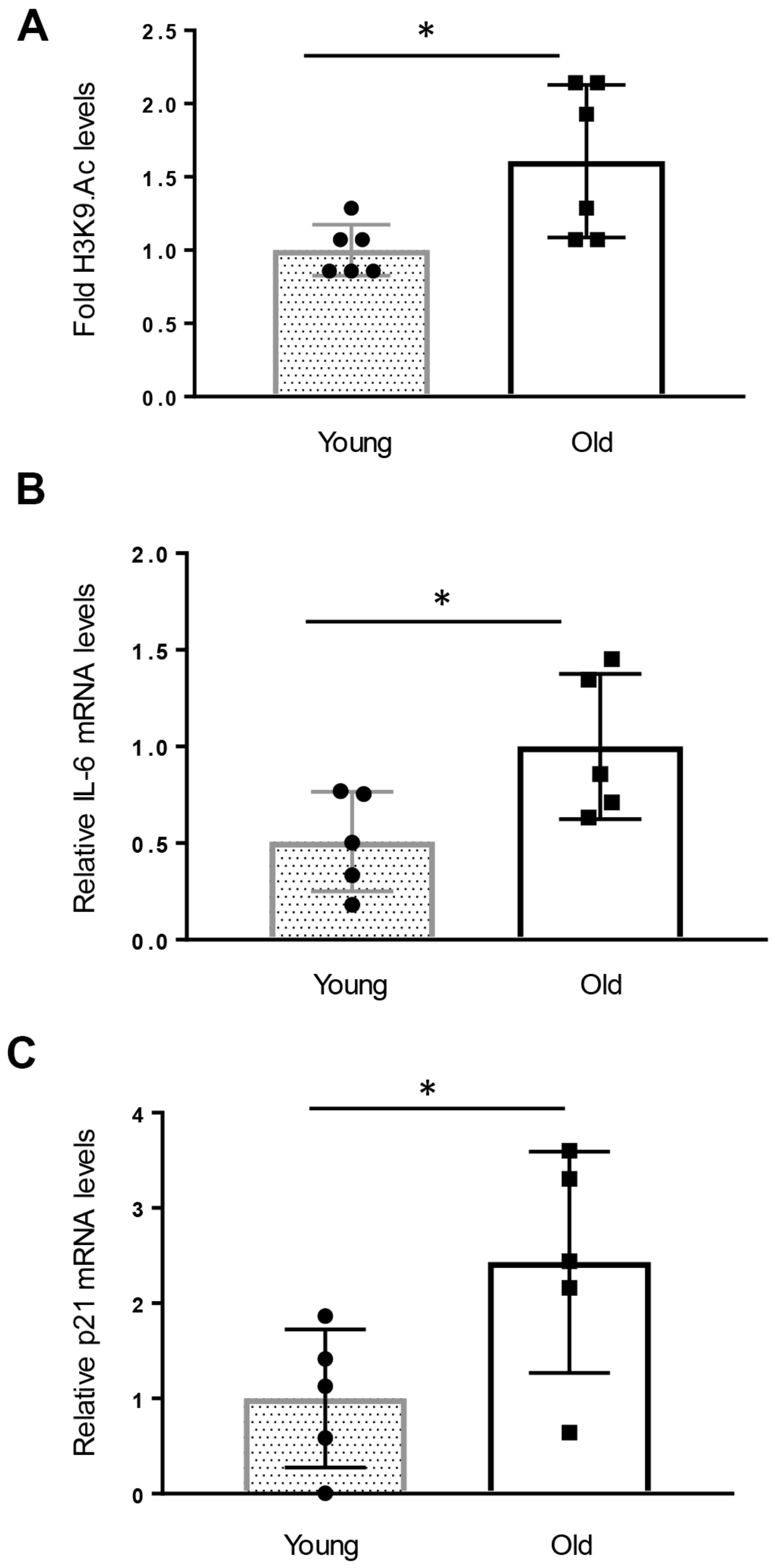

Supplementary Figure 2. Aged mice show increased histone 3 acetylation and increased NF-κB target gene activation: (**A**) Relative acetylated histone H3K9 levels and (**B**) relative Il-6 and (**C**) p21 mRNA levels in the heart of young and old mice. Values are mean ± SE, n = 5-6, * $P < 0.05$.

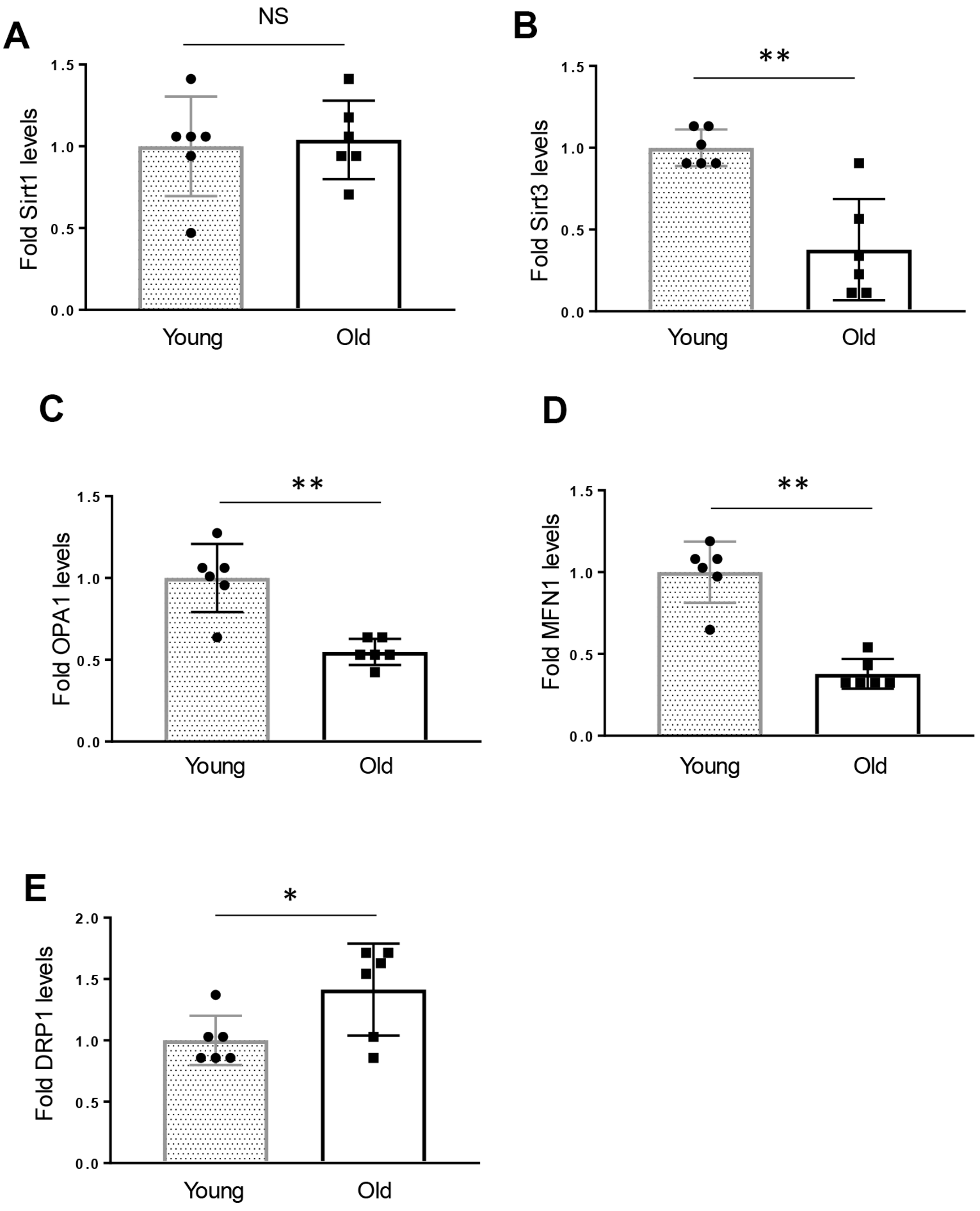

Supplementary Figure 3. Quantification of the western blot in Figure 1F: (**A**–**E**). Quantification of relative Sirt1, Sirt3, OPA1, MFN1 and DRP1 levels in the heart of young and old mice. Values are mean ± SE, n = 6 * P < 0.05, ** P < 0.01 (NS=not significant).

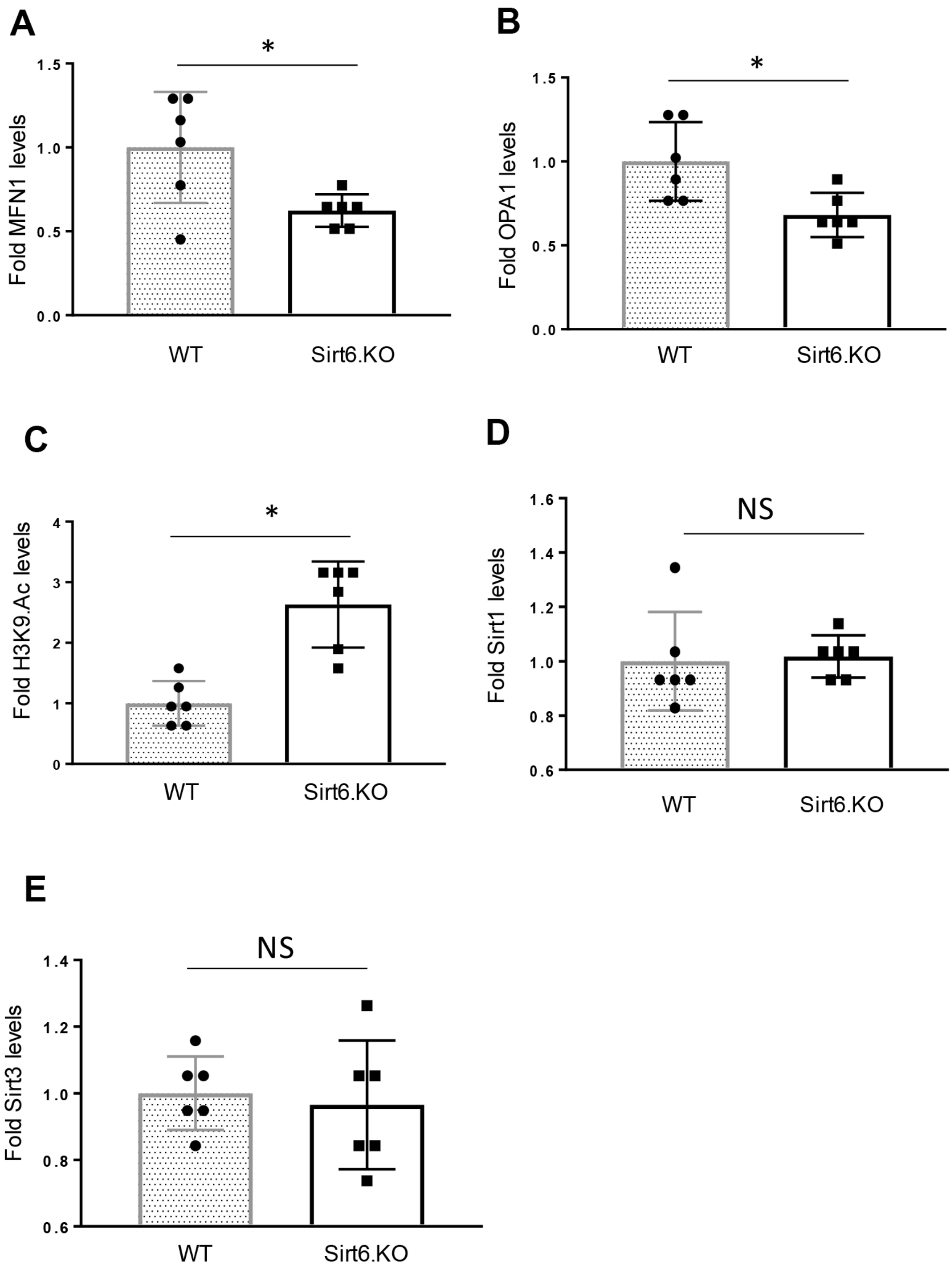

Supplementary Figure 4. Quantification of the western blot in Figure 3F: (**A**–**E**). Quantification of relative MFN1, OPA1, acetylated histone H3K9, Sirt1 and Sirt3 levels in the heart of Wild type and Sirt6.KO mice. Values are mean ± SE, n = 6, * P < 0.01 (NS=not significant).

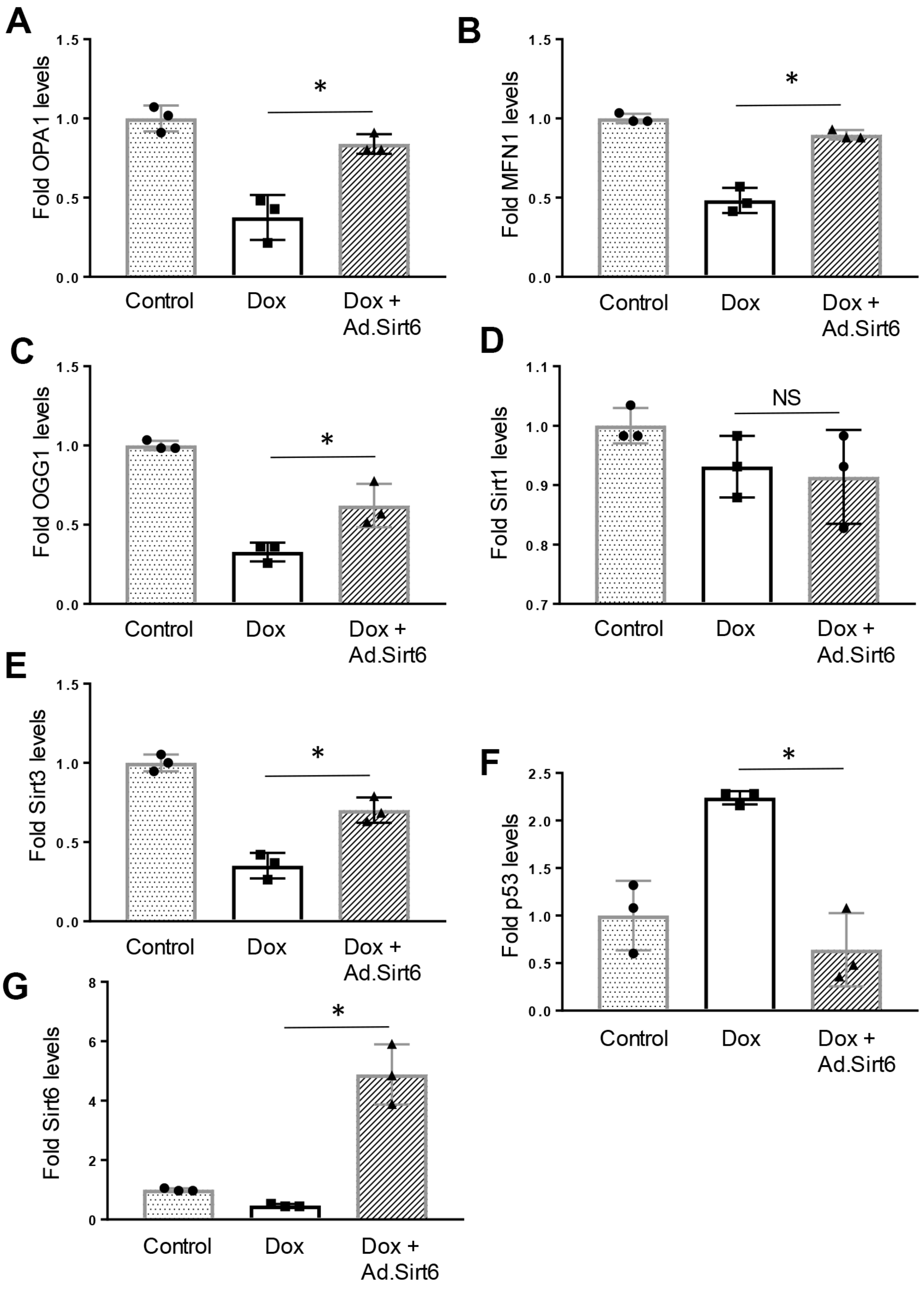

Supplementary Figure 5. Quantification of the western blot in Figure 5C: (**A**–**G**). Quantification of relative OPA1, MFN1, OGG, Sirt1, Sirt3, p53, and Sirt6 levels in cardiomyocytes treated as indicated. Values are average of three independent experiments, mean ± SE, * P < 0.01 (NS=not significant).

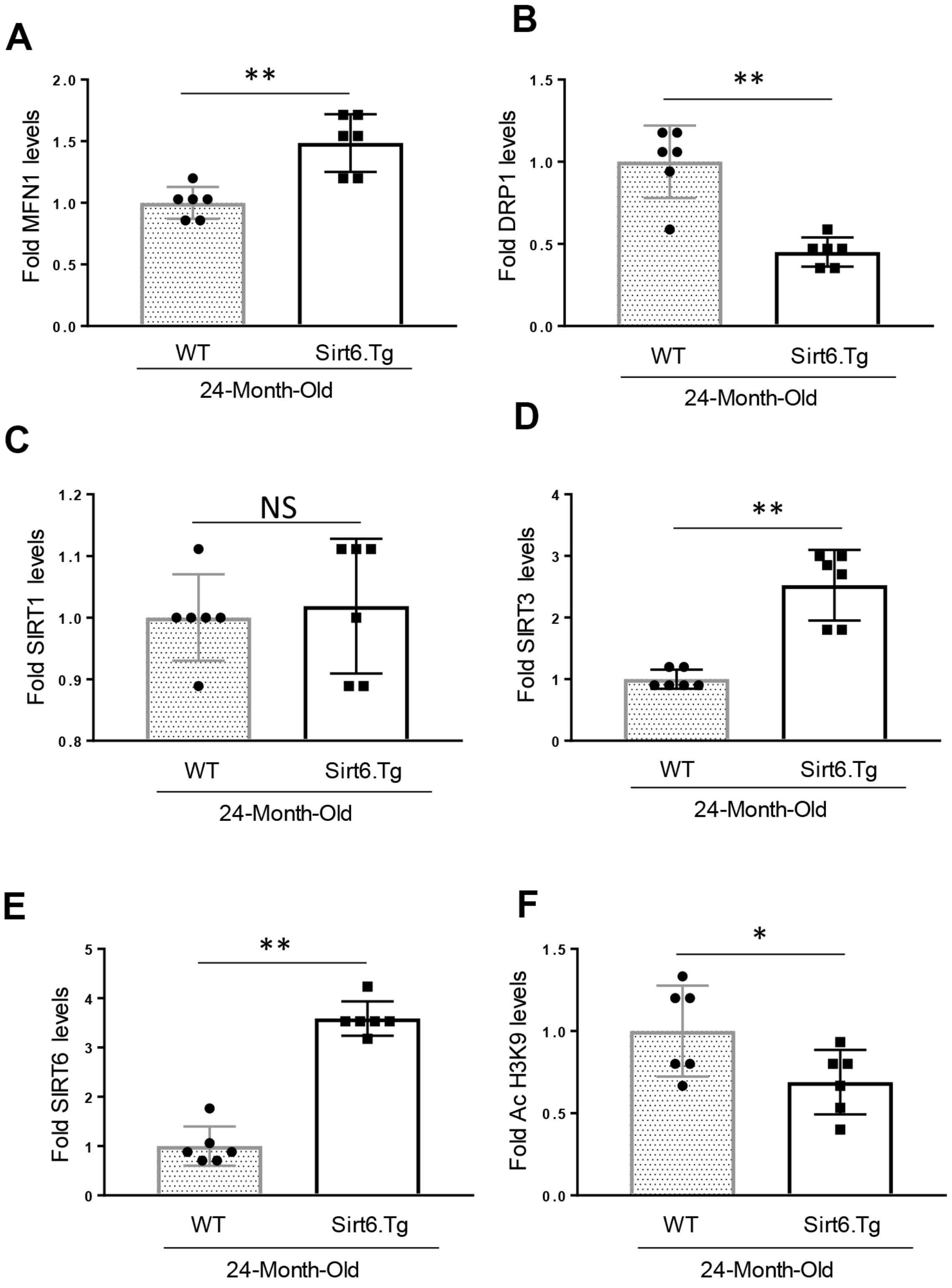

Supplementary Figure 6. Quantification of the western blot in Figure 6E: (**A**–**F**). Quantification of relative MFN1, DRP1, Sirt1, Sirt3, Sirt6, and acetylated histone H3K9 levels in the heart of 24-month-old Wild type and Sirt6.Tg mice. Values are mean ± SE, n = 6, * $P < 0.05$, ** $P < 0.01$ (NS=not significant).
